# Identifying Cellular Stress-Related mRNA Changes Induced by Novel Xanthone Derivatives in Ovarian Cancer Cells In Vitro

**DOI:** 10.3390/pharmaceutics17070816

**Published:** 2025-06-24

**Authors:** Jakub Rech, Dorota Żelaszczyk, Henryk Marona, Ilona Anna Bednarek

**Affiliations:** 1 Department of Biotechnology and Genetic Engineering, Faculty of Pharmaceutical Sciences in Sosnowiec, Medical University of Silesia, 40-055 Katowice, Poland; 2 Department of Bioorganic Chemistry, Chair of Organic Chemistry, Faculty of Pharmacy, Jagiellonian University Medical College, 30-688 Krakow, Poland; dorota.zelaszczyk@uj.edu.pl (D.Ż.); henryk.marona@uj.edu.pl (H.M.)

**Keywords:** xanthones, synthetic xanthone derivatives, chemotherapy, anticancer agents, ovarian cancer therapy, cellular stress response, metastasis, in silico ADME/Tox prediction, heat shock and antioxidant proteins

## Abstract

**Background:** Ovarian cancer is a major challenge in oncology due to high mortality rates, especially in advanced stages, despite current therapeutic approaches relying on chemotherapy and surgery. The search for novel therapeutic strategies is driven by the need for more effective treatments. This study focuses on novel xanthone derivatives modified with a morpholine ring, aiming to improve anticancer efficacy. **Methods:** In silico studies were conducted using ProTox III and SwissADME databases to assess the toxicity and ADME properties of the synthesized compounds. Molecular changes in cellular stress-related genes were investigated through qPCR in two ovarian cancer cell lines (TOV-21G and SKOV-3) following treatment with the compounds. **Results:** In silico analyses predicted high gastrointestinal absorption and blood–brain barrier permeability for the derivatives. Compounds exhibited varying toxicity and metabolic profiles. qPCR revealed significant alterations in genes related to antioxidant enzymes, molecular chaperones, and xenobiotic metabolism, indicating potential mechanisms of action and cellular responses to the compounds. **Conclusions:** The study demonstrates the potential of novel xanthone derivatives as promising candidates for ovarian cancer therapy, with implications for enhancing therapeutic efficacy and addressing drug resistance. Further research is warranted to elucidate the precise mechanisms underlying the observed effects and to develop tailored treatment strategies leveraging these agents.

## 1. Introduction

Ovarian cancer remains a significant challenge in oncology; even despite vast progress in developing new therapies, the majority of new cases are diagnosed in an advanced stage of disease, which results in over 50% mortality [1]. Standard therapies are based on chemotherapy and further surgeon invasion but have shown limited effectiveness, prompting the search for novel therapeutic approaches. New therapies include improved therapeutic delivery, utilization of a combination of agents, and developing new agents, based on well-known compounds and drug delivery systems not only in cancer but also in other diseases. The use of different delivery vehicles is very promising and may benefit from prolonged release time and cell exposure and also a reduction in necessary drug concentration [2,3,4,5].

Such an approach is based on improving therapeutics effectiveness, by introducing small additives to structure, previously examined, with a known mechanism of action and side-effects. These substances may originate from natural products like herbs and fruits but also may be based on modification of synthetic agents. In addition to improving therapeutic effects, this approach may also lead to the development of unique substances to overcome drug-resistance problems in cancer cells. Two examples are α-mangostin (**MAG**) and gambogic acid, both naturally synthesized and found in *Garcinia mangostana* fruits. These agents were found to possess anticancer properties, which results in increased research interest [6]. Moreover, these substances may be used in different cancer-type treatments and are proposed for use in psychiatric disorders like schizophrenia, bipolar disorder, and Alzheimer’s disease [7,8,9].

Throughout its lifespan, the human body is exposed to various stress stimuli, and even slight but recurrent changes in the body’s daily rhythm can lead to gene disruption, potentially resulting in diseases and cancer [10]. Stress proteins responsible for drug metabolism and heat shock proteins are currently targets for anticancer therapies due to their properties. These proteins are involved in xenobiotic metabolism, molecular and cellular responses to it, and environmental stress, thus modulating overall cell survival and drug response [11,12]. This makes them candidates to be included in this study.

**Figure 1:**
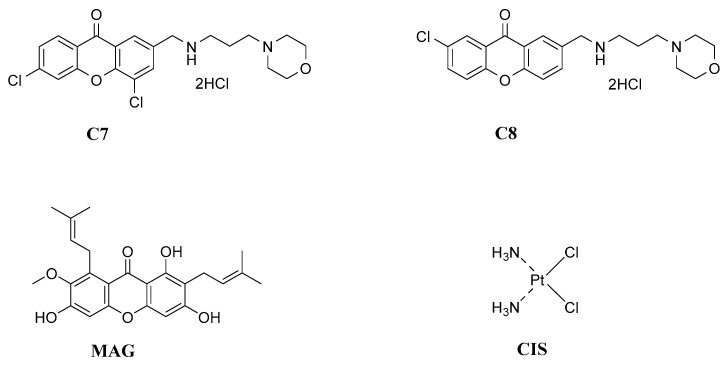
The structure of studied compounds. Both **C7** and **C8** compounds are derivatives of xanthone ring modified with a morpholine ring. **C7**—4,6-Dichloro-2-(((3-morpholinopropyl)amino)methyl)-9*H*-xanthen-9-one Dihydrochloride; **C8**—7-Chloro-2-(((3-morpholinopropyl)amino)methyl)-9*H*-xanthen-9-one Dihydrochloride. Detailed methodology of synthesis was described in [13].

In this paper, we continue our research on two xanthone derivatives (**C7** and **C8**, Figure 1) bearing anticancer efficacy described in our previous studies [13]. 

These derivatives were synthesized based on our previous study where xanthones modified with morpholine moiety were found to exert various biological activities with anticancer activity, which was most important for this study. The additional rationale for choosing these modifications was the increased water solubility of novel xanthone derivatives [7]. These derivatives were chosen from the eight most promising compounds in previous research, based on their overall growth-inhibiting potential toward both of the tested cell lines. We demonstrated growth inhibition activity toward ovarian cancer cells but not toxicity to L929 healthy cells. Additionally, these compounds induced ovarian cancer cell apoptosis, decreased clonogenic potential, and cell motility assessed by wound healing assay. Further studies indicated that compounds influence mitochondria, changing their membrane potential and mass. Also, expression of HSP90A, HSP90B, and Hsc70 was increased under the xanthone treatment [13].

Herein, in silico studies were conducted to validate the potential usage of synthesized derivatives, and further, we describe molecular changes on the expression level of various cellular stress-related genes to unveil the influence of used compounds on two ovarian cancer cell lines with different characteristics.

## 2. Materials and Methods

### 2.1. Chemicals for Biological Evaluations

The studied synthetic xanthone derivatives were as follows: 4,6-dichloro-2-(((3-morpholinopropyl)amino)methyl)-9*H*-xanthen-9-one dihydrochloride (**C7**) and 7-chloro-2-(((3-morpholinopropyl)amino)methyl)-9*H*-xanthen-9-one dihydrochloride (**C8**) were synthesized in the Department of Bioorganic Chemistry, Chair of Organic Chemistry, Faculty of Pharmacy, Jagiellonian University Medical College, Kraków, Poland. Detailed synthesis, and further description and characterisation of derivatives by means of NMR, DSC, and UHPLS/MS; data can be found in [13] and in the Supplemenatry Materials of the mentioned article. **MAG** and cisplatin (**CIS**) were purchased from Sigma-Aldrich (Saint Louis, MO, USA) and Merck KGaA (Darmstadt, Germany) respectively. **CIS** was chosen as a reference as a generally utilized drug for ovarian cancer treatment, and **MAG** as a naturally occurring xanthone reference compound. All IC10 compounds used in the study are summarized in Table 3 in article [13].

Xanthones stock solutions were prepared by dissolving xanthones in DMSO Sigma-Aldrich (Saint Louis, MO, USA) at a concentration of 10 mM each and frozen at −20 °C. **MAG** (24 mM) and **CIS** (10 mM) stock solutions were prepared in methanol (CHEMCALND, Stargard, Poland) and 0.9% NaCl (STANLAB, Lublin, Poland), respectively. Directly before assays, stock solutions were thawed and diluted to the desired concentration in DMEM (ThermoFisher Scientific, Waltham, MA, USA).

### 2.2. Cell Culture and Treatment Conditions

Two ovarian cancer cell lines, namely TOV-21G (ATCC^®^ CRL-11730™) and SKOV-3 (ATCC^®^ HTB-77™), were used in this study. The selection of the tested lines was based on two key factors: their diverse origins and their drug susceptibility characteristics (TOV-21G ovarian clear cell adenocarcinoma, isolated from primary tumor; SKOV-3 ovarian serous cystadenocarcinoma, isolated from ascites; TOV-21G—resistant to **CIS**, SKOV-3 resistant to xanthones) as presented in our previous study [13].

Cells were grown in a standard composition of medium (DMEM High Glucose with L-glutamine (ThermoFisher Scientific, Waltham, MA, USA), 10% FBS (PAN-Biotech GmbH, Aidenbach, Germany), 1% PenStrep (ThermoFisher Scientific, Waltham, MA, USA)) in HeraCell Heraeus (Kendro, Germany) cell incubator at 37 °C and 5% CO_2_.

For assays, cells were prepared by growing overnight, after which removal and addition of fresh media containing xanthones at GI10 for 24 h were conducted. The GI10 values determination results are presented in our previous study in Table 3 [13].

### 2.3. In Silico Analysis

Toxicity analysis of novel xanthone derivatives was conducted in silico by ProTox-III, an online utility for the prediction of toxicity and compound ADMET (absorption, distribution, metabolism, elimination, and toxicity) properties (https://tox.charite.de/, accessed on 15 January 2024). All summarised results are condensed and formatted into a table. Results in bold are concerned as being active toward the respective molecule, and the number value is a probability of impact in the range of 0–1.0.

Further ADME (absorption, distribution, metabolism, and elimination) properties were evaluated with SwissADME online software (http://www.swissadme.ch/, accessed on 15 January 2024).

### 2.4. RNA Extraction and Real-Time RT PCR

Extraction and purification of total RNA was carried out with the GeneMATRIX Universal DNA/RNA/Protein Purification Kit (EUR_X_, Gdańsk, Poland) according to the manufacturer’s protocol. Concentration, quality, and nucleic acid purity ratio (260/280 nm) were measured spectrophotometrically (BioPhotometer, Eppendorf, Warsaw, Poland). Further assessment of extract purity and quality was carried out by band visualization on 2% agarose gels under UV.

Assessment of changes in cellular stress-related mRNA was carried out with RT^2^ Profiler™ PCR Array Human Cellular Stress Responses (GeneGlobe ID: PAHS-019Z, Qiagen, Hilden, Germany) and dedicated kits for first strand synthesis and real-time PCR: RT^2^ First Strand Kit (Qiagen, Hilden, Germany) and RT^2^ SYBR Green ROX qPCR Mastermix (Qiagen, Hilden, Germany), respectively. All procedures were carried out according to manufacturer protocol. Data analysis was performed using dedicated RT^2^ Profiler PCR Data Analysis software available at https://geneglobe.qiagen.com/pl, accessed on 22 February 2024. From housekeeping genes available at the used matrices, we chose Ribosomal protein, large, P0 (RPLP0) as the reference gene for normalisation. The indicated control gene was chosen due to its high expression stability, as reflected by the consistent Ct parameter readings in real-time analyses.

## 3. Results and Discussion

### 3.1. In Silico Studies

As an additive to previous studies, we also conducted two in silico studies to further unveil xanthone’s properties. These were based on ProTox III and SwissADME databases, which compromise prediction data based on trained computer models.

#### 3.1.1. Toxicity Prediction

Initial toxicity studies were propagated in silico with the use of the ProTox-III, an online utility for the estimation of compound properties. The results are summarized in Table 1. Numbers represent probability, but only those in bold and red are selected as active toward the respective properties. Other indistinguished number probabilities indicate inactivity.

**MAG** and **CIS** (Figure 1) were also included in this estimation. Due to the different mechanisms of action of **CIS**, it cannot be directly compared to other compounds. Nevertheless, estimated values of the predicted *o*/*w* coefficient indicate that the chosen compounds are strongly lipophilic with high affinity to cell membranes. Either too high or too low logP values exclude the drug from creating a sustained delivery system, so in this case, we propose to use them on-site, as a one-time infusion after surgery or, e.g., bonded to fibrin gel to additionally prevent internal hemorrhage and enhance healing [2,14].

The tested compounds’ predicted toxicity classes are dependent on predicted LD50 and are class IV which indicates **C8** and **C7** are harmful if swallowed (Class IV—300 < LD50 ≤ 2000). Proper delivery of these compounds via the oral tract would demand appropriate vehicle or encapsulation to diminish cytotoxic activity until targeted cells approach. This predicted data complies with the previously assessed viability of TOV-21G and SKOV-3 cells and their estimated GI50 [13].

**Table 1:** Predicted properties of tested compounds by ProTox-III software.

	Novel Xanthone Derivatives		Control Drug	
	C8	C7	MAG	CIS
**Molecular weight**	385.88	420.33	410.46	259.21
**Octanol/water partition coefficient (logP)**	5.29	5.94	5.09	−1.45
**Predicted LD50 [mg/kg]:**	450	723	1500	200
**Predicted Toxicity Class:**	4	4	4	3
**Hepatotoxicity**	0.77	0.77	0.70	0.72
**Carcinogenicity**	0.55	0.55	0.69	0.63
**Immunotoxicity**	0.64	**0.57**	**0.84**	0.99
**Mutagenicity**	0.58	0.58	0.53	0.56
**Cytotoxicity**	0.53	0.53	0.77	0.72
**Estrogen Receptor Alpha (ER)**	0.79	0.79	0.71	0.97
**Estrogen Receptor Ligand Binding Domain (ER-LBD)**	0.94	0.94	0.82	0.98
**Heat shock factor response element (HSE)**	0.92	0.92	0.81	0.81
**Mitochondrial Membrane Potential (MMP)**	0.88	0.88	**0.67**	0.94
**Phosphoprotein (Tumor Suppressor) p53**	0.92	0.92	0.58	0.96

Data in red is compromised as being “Active”, meaning it is predicted to have an impact on related factors by the presented number value probability. The rest of the data is compromised as inactive by the presented probability.

According to the interpretation of ProTox-III data, only **C8** indicated no toxic behavior, and **C7** and **MAG** are predicted to be immunotoxic (probability 0.57 and 0.84, respectively). Current knowledge describes no direct immunotoxicity, but we found papers stating the regulating effect of **MAG** toward inflammation, PI3K/Akt/mTOR pathway, and in vitro apoptosis studies involving various leukemia cells and healthy human peripheral blood leucocytes where **MAG** had a significant effect in low concentrations (~10 μM) [15,16].

#### 3.1.2. ADME Properties

Further investigation of in silico properties of selected xanthone derivatives included ADME properties description, which are summarized in Table 2.

All selected xanthone derivatives are supposed to be highly absorbed in the intestine (GI absorption value) and permeant for the blood–brain barrier. Such compounds could be easily administered by oral tract, but compared with logP value (Section 3.1.1), this activity needs further attention and research.

Additionally, **C8** and **C7** are predicted to be a substrate for P-gp, thus being actively expelled from, e.g., the CNS system, protecting it from xenobiotics. This research is targeted toward ovarian cancer cell lines, but high P-gp activity is considered to be responsible for drug resistance especially in cancer cell lines [17].

Reference compounds, **MAG**, and **CIS** are predicted not to be BBB-permeant and P-gp substrates and also to have no influence on CYP isoforms, excluding **MAG** and CYP2C9. This complies with their empirically assessed properties.

Druglikeness properties are calculated according to five different approaches—Lipinski, Ghose, Veber, Egan, and Muegge. All selected xanthone derivatives are estimated to be good candidates for oral drugs. The calculated bioavailability score for selected compounds is enough to select these compounds for further drug-screening process.

Only violations of druglikeness occurred for **MAG** and **CIS**, and are related to their small size (Ghose, **CIS**), high logP (Muegge, **MAG**), or low carbon atoms count (Muegge—**CIS**).

**Table 2:** ADME properties of selected compounds estimated by SwissADME software.

	Novel Xanthone Derivatives		Control Drug	
	C8	C7	MAG	CIS
	Pharmacokinetics			
GI absorption	High	High	High	High
BBB permeant	Yes	Yes	No	No
P-gp substrate	Yes	Yes	No	No
CYP1A2 inhibitor	Yes	Yes	No	No
CYP2C19 inhibitor	Yes	Yes	No	No
CYP2C9 inhibitor	Yes	Yes	Yes	No
CYP2D6 inhibitor	Yes	Yes	No	No
CYP3A4 inhibitor	Yes	Yes	No	No
**Druglikeness Criteria**				
Lipinski	Yes	Yes	Yes	Yes
Ghose	Yes	Yes	Yes	No *
Veber	Yes	Yes	Yes	Yes
Egan	Yes	Yes	Yes	Yes
Muegge	Yes	Yes	No **	No ***
Bioavailability Score	0.55	0.55	0.55	0.55

GI—gastrointestinal, BBB—blood–brain barrier, P-gp—permeability glycoprotein, * 3 violations: WLOGP < −0.4, MR < 40, number of atoms < 20; ** 1 violation: XLOGP3 > 5; *** 1 violation: number of C atoms < 5.

### 3.2. qPCR of Cellular Stress-Related Proteins

To estimate the influence of tested compounds on human cellular stress responses, designated RT^2^ Profiler™ PCR Arrays were used. These arrays contain 84 genes, divided into antioxidant and pro-oxidant enzymes categories; the molecular chaperones category, including heat shock proteins and other molecular chaperones; the xenobiotic metabolism category, including cytochrome p450s isoforms; and other xenobiotic metabolism gene categories. A detailed gene list is described in Table 3.

Genes whose regulation fold was above 2 or below −2 were considered significant and further discussed. Nevertheless, genes with regulation fold, close to the set threshold but not exceeding it, are also mentioned, due to the possible influence of tested compounds.

#### 3.2.1. Antioxidant and Pro-Oxidant Enzymes

**Figure 2:**
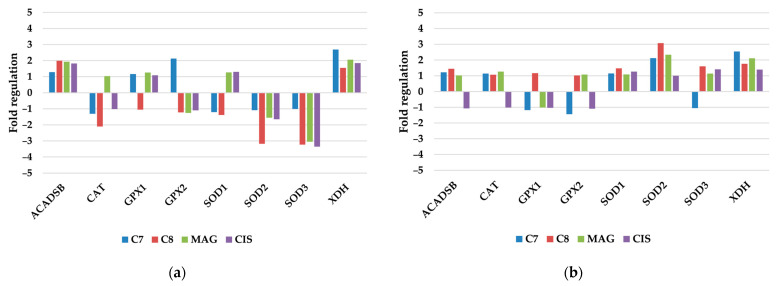
Regulation fold of antioxidant and pro-oxidant enzyme genes of (**a**) TOV-21G and (**b**) SKOV-3 cells treated with investigated compounds.

Out of the eight tested reactive oxygen species (ROS)-related genes, the expression change in three of them was significant. Mentioned are SOD2, SOD3, and XDH, all of which are associated with the removal of ROS. Additionally, different results were obtained in the case of TOV-21G and SKOV-3 cell lines (Figure 2).

In TOV-21G, SOD2 and SOD3 were downregulated, mostly by **C8**. Conversely, SOD2 and 3 (not significant) were upregulated in SKOV-3 cells. XDH in both cases was upregulated with the highest influence of **C7** and **MAG**. Xanthone derivatives’ anticancer mechanisms of action are supposed to be related to cellular ROS level modification, resulting in irreversible damage to cell compartments. These results are supported by our previous research on colon cancer cells [18]. Inhibition of dismutase expression and further decreased ability to remove ROS could result in much higher levels of ROS presence in cells and increased cell damage and death [19]. This phenomenon may be exerted by TOV-21G cells, in which tested compounds hampered the expression of SOD2, -3, and XDH, while SKOV-3 levels of mentioned genes were increased. Nevertheless, the mode of action strongly depends on the xanthone derivative structure due to the fact that these compounds may also act as ROS scavengers [20,21]. This ROS scavenging activity seems to be connected with a hydrogenated xanthone ring, without large scaffolds, such as our morpholine-ring-modified xanthones or triphenylphosphonium-cation-moiety-introduced xanthones [22].

Also, TOV-21G cells increased expression of GPX2 while treated with **C7** and significantly decreased expression of CAT while treated with **C8**. These two genes (glutathione peroxidase 2 and catalase, respectively) are also involved in ROS removal and their change in expression may also indicate an increased level of free radicals in cells [18,19].

#### 3.2.2. Xenobiotic Metabolism—p450

This group of genes consists of different isoforms of cytochrome p450 that are considered the most important enzymes involved in xenobiotic metabolism.

**Figure 3:**
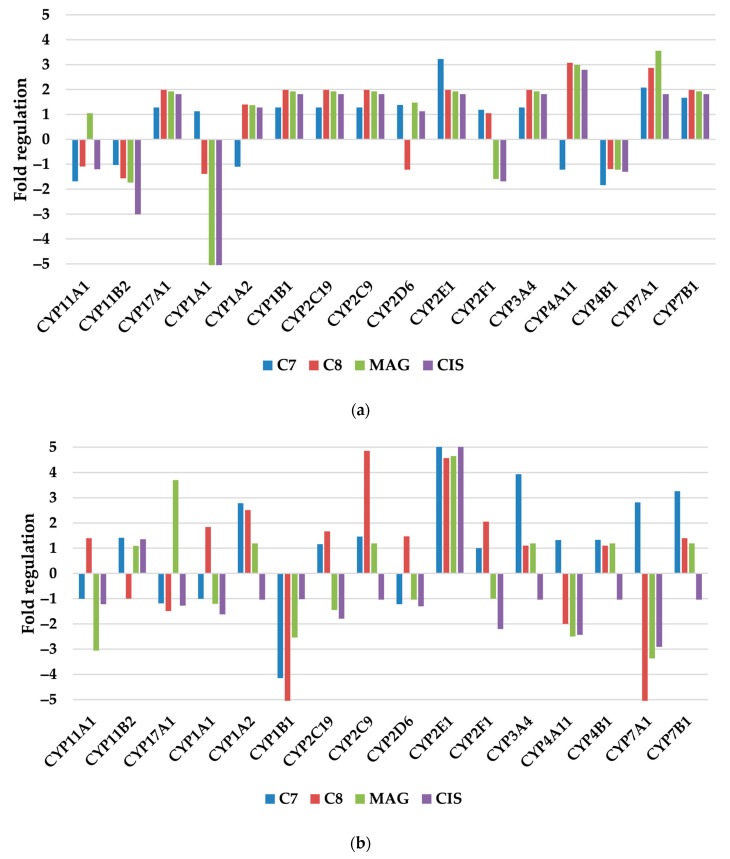
The regulation fold of xenobiotic metabolism—p450 isoforms genes of (**a**) TOV-21G and (**b**) SKOV-3 cells treated with investigated compounds. Gene expression values were cropped to a range of −5 to 5 to ensure appropriate data presentation. Excessing TOV-21G CYP1A1 fold regulation: **MAG** −5.5; **CIS** −5.86. Excessing SKOV-3 CYP1B1 fold regulation: **C8** −9.33; CYP2E1 **C7** 5.6; **CIS** 14.42. CYP7A1 **C8** −10.21.

TOV-21G cells response was more delicate and predictable; generally, all tested substances had a similar impact toward one isoenzyme, either increasing or decreasing expression (Figure 3). The cytochrome p450 isoenzymes (CYPs) that were the most influenced are 1A1, 4A11, 7A1, 11B2, and 2E1. CYP1A1 expression was greatly hampered to −5.5 and −5.86 fold by **MAG** and **CIS**, respectively. **CIS** also inhibited the expression of CYP11B2 to about −3x fold. Xanthones generally increased most of the p450 isoenzymes, with the greatest impact on CYP2E1 (**C8**), CYP4A11 (**C7**, **MAG**), and CYP7A1 (**C7**, **MAG**).

SKOV-3 presented different results, more consistent in the case of **CIS**, which mostly inhibited the expression of all the CYP’s but not in a relevant manner. Isoenzymes that exerted significant response are as follows: CYP11A1 (**MAG**), CYP17A1 (**MAG**), CYP1A2 (**C8**, **C7**), CYP1B1 (**C8**, **C7**, **MAG**), CYP2C9 (**C8**), CYP2E1(ALL), CYP2F1(**C8**, **CIS**), CYP3A4(**C7**), CYP4A11 (**C8**, **MAG**, **CIS**), CYP 7A1 (**C8**, **MAG**, **CIS**), and CYP7B1(**C7**). The greatest impact was on CYP7A1 (−10.21; **C8**) and CYP2E1 (14.42; **CIS**).

On both cell lines, isoenzymes CYP2E1 (**C7**), CYP4A11 (**C8**, **MAG**, **CIS**), and CYP7A1 (**C8**, **MAG**) expression changed under treatment of substances in brackets, respectively.

Out of many cytochrome isoforms, the activity of all of them revolves around metabolizing xenobiotics. The most downregulated expression was recorded for two isoforms, namely CYP1A1 and CYP1B1. The first mentioned contributes to the transformation of xenobiotics with procancerous properties into active ones, and its downregulation may be connected with slower cancer progression. The latter, CYP1B1, was found to be overexpressed in the majority of ovarian cancers, whereas no elevation was found in healthy tissues [23]. Both of them are regulated by aryl hydrocarbon receptor (AhR), which, in response to xenobiotics, activates CYP1A1 and CYP1B1, which leads to the conversion of procarcinogens into ultimate carcinogens. In ovarian cancers, this pathway activates the PI3K/AKT pathway, with activation of mesenchymal–epithelial transition and ongoing metastasis [24].

CYP2E1 is linked with cancer, and its higher activity was found in sera of patients with ovarian cancer. It is also correlated with upregulated levels of the proinflammatory proteins IL-6, IL-8, and TNF-α [25].

CYP7A1 is the main enzyme responsible for bile acids synthesis and it is a step-limiting enzyme with negative feedback regulation from bile acids. Different concentrations of these compounds detected in patients with ovarian cancer were proposed as markers in screening for this disease [26].

#### 3.2.3. Other Xenobiotic Metabolism Genes

**Figure 4:**
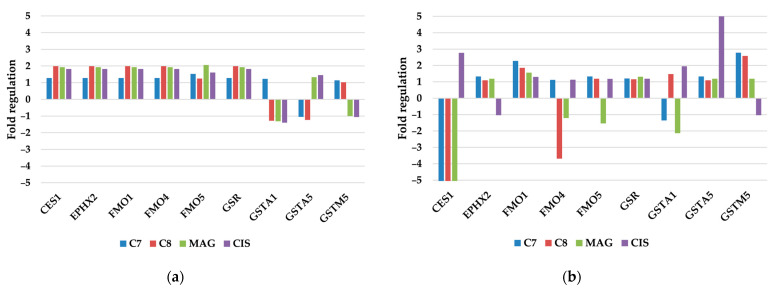
The regulation fold of other xenobiotic metabolism genes of (**a**) TOV-21G and (**b**) SKOV-3 cells treated with investigated compounds. Gene expression values were cropped to a range of −5 to 5 to ensure appropriate data presentation. Excessing SKOV-3 CES1 fold regulation: **C7** −5.08; **C8** −6.16; **MAG** −5.71.

In other xenobiotic metabolism gene groups, only SKOV-3 cells showed a response to drug treatment (Figure 4). 

CES1 expression was hampered to the levels of −5.08, −6.16, and −5.71 by **C7**, **C8**, and **MAG,** respectively. **CIS** treatment, on the contrary, promoted expression to 2.77. This gene encodes carboxylesterase 1 protein, responsible for the detoxification of xenobiotics, containing ester or amine bonds that are not present in our compounds. Strong impairing activity toward the expression of the CES1 gene of xanthones may further increase its toxicity in SKOV cells. CES is also investigated to be responsible for the activation of prodrugs (like Ironotecan); thus, a lack of activity may lead to additional efforts of CYP isoenzymes to detoxify and inactivate used compounds [27]. Other studies indicate that the CES1 role is not only restricted to prodrug activation, but these enzymes take part in lipid metabolism and a decrease in activity was discovered in erastin-induced ferroptosis [28]. This is another clue that the SKOV cell’s death mechanism was ferroptotic.

**C8** also hampered the expression of the FM04 (dimethylaniline monooxygenase 4) enzyme, which is linked to the catabolism of nucleophilic heteroatom centers in drugs and is connected with drug resistance and ROS removal [29,30]. Another group of influenced enzymes were glutathione S-transferase (GSTA) enzymes involved in adding glutathione to various compounds, detoxifying them, and removing ROS—which is another clue that xanthone’s mode of action is connected with modulating ROS [31]. GSTA1 was only significantly modulated by **MAG** (inhibition to −2.13x fold). GSTA5 expression was increased by **CIS** (6.28x fold), and GSTM5 expression was increased after **C7** and **C8** treatment to 2.78 and 2.58, respectively.

#### 3.2.4. Molecular Chaperones

Heat shock proteins (HSPs) are a group of molecular chaperones, present in both healthy and cancer cells. Their main functions are connected with a reaction to cellular stress generated by extracellular factors, which may lead to modulation of immune response and angiogenesis or changes in cell metabolism and further apoptosis. All mentioned processes are strictly involved in cancer development; thus, currently, these proteins are in the scope of many researchers, searching for appropriate targets within extensive HSPs both in vitro and in vivo [11,12].

#### 3.2.5. Heat Shock Proteins

Heat shock proteins are a large group of chaperone proteins, serving as defenders against stressful agents and regulating cell metabolism to keep homeostasis. Hsp90, Hsp70, and Hsp40 are regarded as the most important families. These families consist of various proteins having a common influence on cancer cells, thus being proposed as biomarkers in ovarian, breast, and many other cancer types. Additionally, these proteins have become new targets for emerging anticancer drugs.

**Figure 5:**
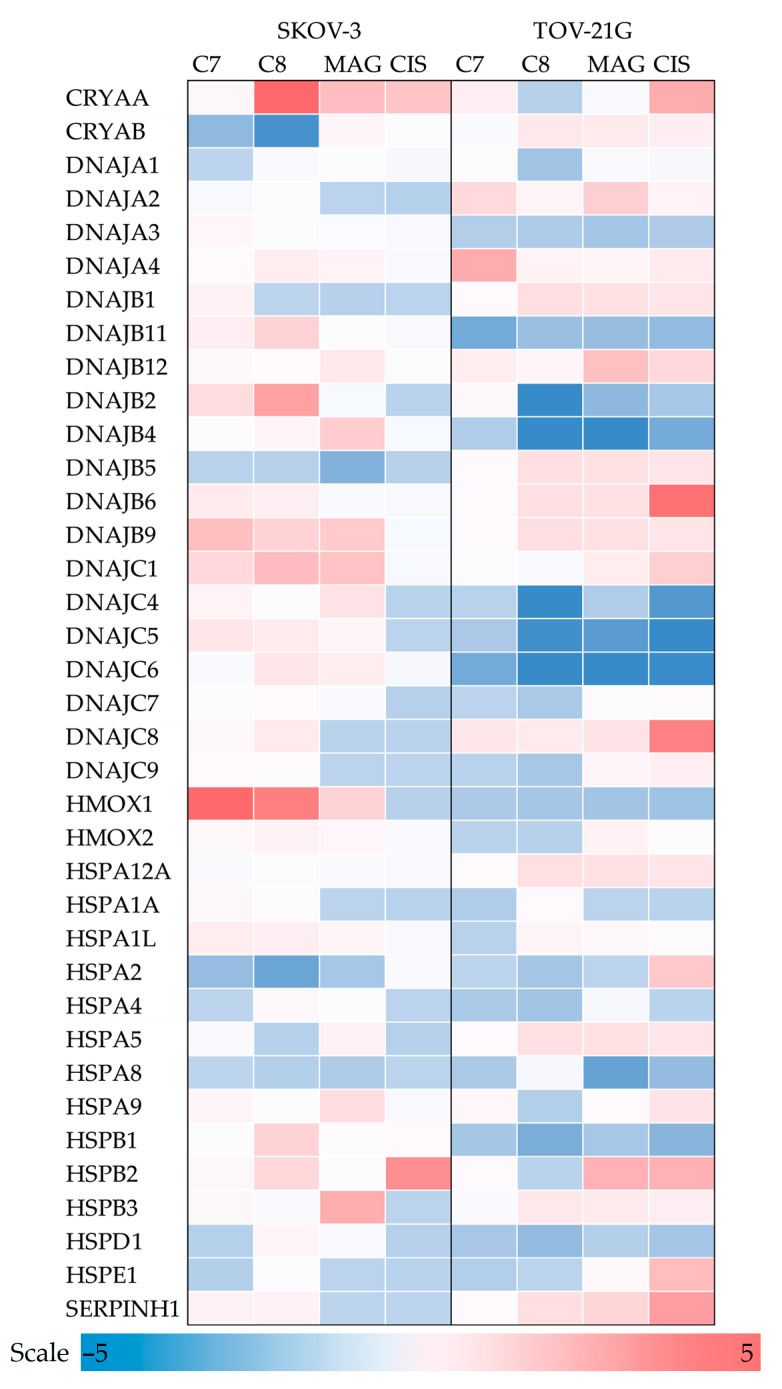
The regulation fold of heat shock proteins genes of TOV-21G and SKOV-3 cells treated with investigated compounds. Gene expression values were cropped to a range of −5 to 5 to ensure appropriate data presentation. Excessing TOV-21G fold regulation: DNAJB2 **C7** −5.82; DNAJB4 **C8** −6.15; **MAG** −5.74; DNAJC4 **C8** −6.23; DNAJC5 **CIS** −5.74; DNAJC6 C9 −6.82; **MAG** −9.99. Excessing SKOV-3 fold regulation: CRYAA **C8** 6.01; HMOX1 **C7** 6.85.

In this study, we investigated 37 HSPs, out of which 20 for TOV-21G and 13 for SKOV-3 were deregulated (Figure 5). The influence of tested compounds was higher on **C8**-treated TOV-21G cells, where it reached −5.82, −6.15, −6.23, −4.72, and −6.82 for DNAJB2, DNAJB4, DNAJC4, DNAJC5, and DNAJC6, respectively. Such changes were not as spectacular for **C8**-treated SKOV-3 cells, where the highest impact of 6.01 and −4.6 were obtained for CRYAA and CRYAB, respectively. Also, SKOV-3 cells presented elevated XMOX1 expression to 6.85 and 4.49 after **C7** and **C8** treatment, respectively. Additionally, comparable dysregulation patterns between the cell lines were only presented by HSPA2.

CRYAA and CRYAB proteins belong to the small heat shock proteins (Hsp20) family. Their mode of action is slightly different than classic chaperones. Instead of refolding denatured proteins, they hold these proteins in large soluble aggregates, decreasing their unpleasant influence on cells. Increased expression of CRYAB has been linked with poor prognosis due to the promotion of survival and diminishing of apoptosis. In the case of ovarian cancer, elevated levels of both CRYAB and p53 are regarded as bad prognosis factors and are linked with additional **CIS**-induced apoptosis resistance [32].

DNAJB2, DNAJB4, DNAJC4, DNAJC5, and DNAJC6 belong to the Hsp40 family and are known as co-chaperones, regulators of Hsp70, and also influencing Hsp90. The effects of the action of these proteins may be anti- or pro-cancerous, due to the fact that they have regulating functions in various cells [33]. Increased expression of Hsp40 proteins may stimulate Hsp70 activity, but in our studies, the expression of Hsp70 members (HSPA1A, HSPA4) was not influenced significantly [34].

DNAJB11 gene expression is described as correlated with multiple drug resistance including paclitaxel and **CIS** resistance [35]. Previously calculated GI50 of TOV-21G and SKOV-3 cells does not differ drastically, so this may be the direct effect of the drugs used [13]. Low levels of DNAJB4 are considered a good prognostic marker, indicating decreased metastasis and tumor growth in colorectal and lung cancers [36]. After drug exposure, in TOV-21G cells, these effects were obtained, which is in correlation with previously described wound healing assay results of our studies [13]. What should be considered is SKOV-3 DNAJB4’s low impact and expression change, but in comparison to the previously mentioned research, the overall impact on migration, metastasis, and malignancy was considered significant.

DNAJC members are considered to be carriers of specialized proteins to HSP70 machinery. Nevertheless, their involvement in cancer development is not on a satisfactory level and is still progressively under research [32].

Elevated SKOV-3 HMOX1 levels, in combination with elevated levels of SOD2, may indicate ferroptosis induced by **C7** and **C8**, but complete confirmation should also include enzymes responsible for lipid peroxidation and glutathione peroxidase. In this study, we measured GPX1 and GPX2 expression, which was not elevated (except **C7** treated TOV-21G), but the GPX4 isoform was concluded to be involved in ferroptosis [37]. Induction of ferroptosis can be used for further **CIS** resistance omitting an increase in clinical output [38]. On the other hand, SOD1 and SOD2 lowered expression in TOV-21G cells, which may lower the possibility of ROS accumulation and thus ferroptosis occurrence, but to prove it, another study should be conducted.

#### 3.2.6. Other Molecular Chaperones

**Figure 6:**
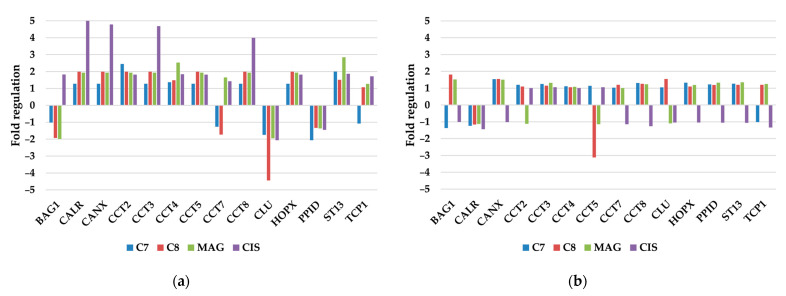
Regulation fold of other molecular chaperone genes of (**a**) TOV-21G and (**b**) SKOV-3 cells treated with investigated compounds.

Other molecular chaperone gene changes were generally affected by **CIS** in TOV-21G cells (CALR, CANX, CCT3, CCT8; Figure 6). CALR encodes calreticulin—a multifunctional protein involved in various cellular processes and calcium storage. Its upregulation may be profitable by increasing tumor immunogenicity by increased MHC class I particle exposure [39]. This protein cooperates with CANX—another protein taking part in calcium storage and protein chaperoning in the so-called CALR/CANX cycle. Similar to CALR, CANX is also responsible for MHC class I antigen presentation [40].

CCT3 and CCT8 both belong to the chaperonin-containing TCP1 family, a group of chaperonin proteins, which are dysregulated in various cancer types [41]. Suppression of the expression of CCT3 was indicated to be the main reason for ROS level increase, mitochondrial membrane decrease, and apoptosis induction in breast and prostate cells [42].

Additionally, **C8** strongly reduced the expression of the CLU gene, which has many contradictory functions depending on its nuclear or secretory isoform [43]. Most of the research points out that suppression of the expression of CLU may inhibit tumor progression and invasiveness, induce apoptosis, and lower chemoresistance of ovarian cancer cells [44].

SKOV-3 cells were more resistant to tested compounds, and generally, the gene expression ratio was not influenced. Only **C8** had a reducing influence on CCT5 gene expression.

This study describes the activity of two novel morpholine-ring-modified xanthones in ovarian cancer treatment. Summarizing the results suggests that these derivatives’ main mode of action is revolving around ROS-related-enzyme expression modulation (**C7**, **C8**, **MAG**), HSP expression dysregulation (**C7**, **C8**), xenobiotic detoxification enzyme dysregulation (**C7**, **C8**, **MAG**, **CIS**), and possible activation of ferroptosis (**C7**, **C8**).

Changes in HMOX1, CES1, and GPX2 expression, especially in SKOV-3, may indicate ferroptosis activation, different from the apoptosis mechanism of cell death. This mechanism may be especially promising for **CIS**-resistant cells and indicate further areas of research toward combined and simultaneous use of **CIS** and novel xanthone derivatives in treatment. An additional claim is that ROS production, CYPs, HSP, and ferroptosis induction are synergistic with **CIS** activity, thus further enhancing their activity [45].

One of the most frequently reported dysregulated pathways in ovarian cancer is the PI3K/AKT/mTOR pathway. Genes like CAT, GPX1, SOD1, and SOD2 are regulated by Akt, indicating an impact on PI3K/AKT pathway, which should be further investigated [46].

Another suggested pathway is the unfolded protein response (UPR)/endoplasmic reticulum (ER) stress pathway. In our opinion, endoplasmic reticulum (ER)/UPR stress can be the main mechanism of action of compounds. Disruption of HSP expression has a direct impact on proper protein folding, especially when cells are exposed to stress. Previously described changes in mitochondria membrane potential and mass, possible ROS generation, increased apoptosis, and possible ferroptosis are all connected with ER stress and can be regulated by it [47]. This is only a hypothesis that should be further developed and supported by studies on key ER regulators, ROS quantification, and apoptotic and ferroptotic protein level estimation.

## 4. Conclusions

Ovarian cancer remains a formidable clinical challenge necessitating the development of innovative treatment strategies. Novel xanthone derivatives offer a promising solution for improving therapeutic efficacy and overcoming drug resistance in ovarian cancer. Additional benefits can be obtained by chemical modification of known structures, increasing their activity. These data suggest that the main mechanism of action of the drugs used is targeted toward mitochondria and ferroptosis. These data should be further supported by protein-level research. Future studies are essential for in-depth unveiling of the precise mechanism of action of used compounds as well as developing new strategies of treatment based on these agents.

## Figures and Tables

**Table 3:** Detailed list of genes of interest, divided into groups.

Antioxidant and Pro-Oxidant Enzymes
ACADSB, CAT, GPX1, GPX2, SOD1, SOD2, SOD3, XDH
**Molecular Chaperones**
**Heat Shock Proteins**
CRYAA, CRYAB, DNAJA1, DNAJA2, DNAJA3, DNAJA4, DNAJB1, DNAJB11, DNAJB12, DNAJB2, DNAJB4, DNAJB5, DNAJB6, DNAJB9, DNAJC1, DNAJC4, DNAJC5, DNAJC6, DNAJC7, DNAJC8, DNAJC9, HMOX1, HMOX2, HSPA12A, HSPA1A (HSP70-1A), HSPA1L, HSPA2, HSPA4(HSP70), HSPA5 (GRP78), HSPA8, HSPA9, HSPB1 (HSP27), HSPB2, HSPB3, HSPD1, HSPE1, SERPINH1 (HSP47)
**Other Molecular Chaperones** BAG1, CALR, CANX, CCT2, CCT3, CCT4, CCT5, CCT7, CCT8, CLU, HOPX, PPID, ST13, TCP1
**Xenobiotic Metabolism**
**Cytochrome P450s**
CYP11A1, CYP11B2, CYP17A1, CYP1A1, CYP1A2, CYP1B1, CYP2C19, CYP2C9, CYP2D6, CYP2E1, CYP2F1, CYP3A4, CYP4A11, CYP4B1, CYP7A1, CYP7B1
**Other Xenobiotic Metabolism Genes**
CES1, EPHX2, FMO1, FMO4, FMO5, GSR, GSTA1, GSTA5 (YC2), GSTM5

## Data Availability

The data presented in this study are available upon reasonable request from the corresponding author.

## Abbreviations

The following abbreviations are used in this manuscript:

ADMET	Absorption, distribution, metabolism, elimination, toxicity
ADME	Absorption, distribution, metabolism, elimination
AhR	Hydrocarbon receptor
BBB	Blood–brain barrier
**CIS**	Cisplatin
CYP	Cytochrome p450 isoenzyme
GI	Gastrointestinal
**MAG**	α-Mangostin
P-gp	Permeability glycoprotein
ROS	Reactive oxygen species
